# Chitotriosidase 1 in the cerebrospinal fluid as a putative biomarker for HTLV-1-associated myelopathy/tropical spastic paraparesis (HAM/TSP) progression

**DOI:** 10.3389/fimmu.2022.949516

**Published:** 2022-08-16

**Authors:** Yago Côrtes Pinheiro Gomes, Nicole Lardini Freitas, Flávia Santos Souza, Vanessa Sandim, Denise Abreu Pereira, Fábio César Sousa Nogueira, Juliana Echevarria-Lima, Ana Claudia Celestino Bezerra Leite, Marco Antonio Sales Dantas Lima, Marcus Tulius Teixeira Silva, Abelardo Queiroz Campos Araújo, Ana Carolina Paulo Vicente, Otávio Melo Espíndola

**Affiliations:** ^1^ Evandro Chagas National Institute of Infectious Diseases (INI), Oswaldo Cruz Foundation (FIOCRUZ), Rio de Janeiro, Brazil; ^2^ Oswaldo Cruz Institute (IOC), Oswaldo Cruz Foundation (FIOCRUZ), Rio de Janeiro, Brazil; ^3^ Institute of Medical Biochemistry Leopoldo de Meis (IBqM), Federal University of Rio de Janeiro (UFRJ), Rio de Janeiro, Brazil; ^4^ Program of Cellular and Molecular Oncobiology (POCM), National Institute of Cancer (INCA), Rio de Janeiro, Brazil; ^5^ Laboratory of Proteomics, Laboratory for the Support of Technological Development (LADETEC), Institute of Chemistry, Federal University of Rio de Janeiro (UFRJ), Rio de Janeiro, Brazil; ^6^ Proteomics Unit, Institute of Chemistry, Federal University of Rio de Janeiro (UFRJ), Rio de Janeiro, Brazil; ^7^ Institute of Microbiology Paulo de Góes, Federal University of Rio de Janeiro (UFRJ), Rio de Janeiro, Brazil

**Keywords:** HTLV-1, HAM/TSP, biomarkers, cerebrospinal fluid, neurodegeneration, chitotriosidase 1, soluble VCAM-1, proteomic analysis

## Abstract

Human T-lymphotropic virus type 1 (HTLV-1)-associated myelopathy/tropical spastic paraparesis (HAM/TSP) is an inflammatory neurodegenerative disease that affects motor, urinary, intestinal, and sensory functions. Typically, HAM/TSP is slowly progressive, but it may vary from limited motor disability after decades (very slow progression) to loss of motor function in a few years from disease onset (rapid). In this study, we aimed to identify prognostic biomarkers for HAM/TSP to support patient management. Thus, proteomic analysis of the cerebrospinal fluid (CSF) was performed with samples from HTLV-1 asymptomatic carriers (AC) (n=13) and HAM/TSP patients (n=21) with rapid, typical, and very slow progression using quantitative label-free liquid chromatography/tandem mass spectrometry. Enrichment analyses were also carried out to identify key biological processes associated with distinct neurological conditions in HTLV-1 infection. Candidate biomarkers were validated by ELISA in paired CSF and serum samples, and samples from HTLV-1-seronegative individuals (n=9) were used as controls. CSF analysis identified 602 proteins. Leukocyte/cell activation, immune response processes and neurodegeneration pathways were enriched in rapid progressors. Conversely, HTLV-1 AC and HAM/TSP patients with typical and very slow progression had enriched processes for nervous system development. Differential expression analysis showed that soluble vascular cell adhesion molecule 1 (sVCAM-1), chitotriosidase 1 (CHIT1), and cathepsin C (CTSC) were upregulated in HAM/TSP. However, only CHIT1 was significantly elevated after validation, particularly in HAM/TSP rapid progressors. In contrast, none of these biomarkers were altered in serum. Additionally, CSF CHIT1 levels in HAM/TSP patients positively correlated with the speed of HAM/TSP progression, defined as points in the IPEC-2 HAM/TSP disability scale per year of disease, and with CSF levels of phosphorylated neurofilament heavy chain, neopterin, CXCL5, CXCL10, and CXCL11. In conclusion, higher CSF levels of CHIT1 were associated with HAM/TSP rapid progression and correlated with other biomarkers of neuroinflammation and neurodegeneration. Therefore, we propose CHIT1 as an additional or alternative CSF biomarker to identify HAM/TSP patients with a worse prognosis.

## 1 Introduction

The human T-lymphotropic virus (HTLV) type 1 infection is associated with the development of two main diseases: a malignancy of CD4^+^ T-cells named adult T-cell leukemia/lymphoma (ATLL) and the HTLV-1-associated myelopathy/tropical spastic paraparesis (HAM/TSP) (1, 2), which is a chronic inflammatory neurodegenerative disease that affects mainly the upper motor neurons in the thoracic spinal cord (3, 4). HTLV-1 infection is worldwide distributed but it shows hot spots of endemicity in several countries and regions, including Brazil, Japan, the Caribbean, Central and West Africa, the Middle Eastern, and Australia (5–9). In recent years, HTLV-1 infection has been also considered a matter of concern as it remains a threat to communities, particularly in low-income countries due to the lack of epidemiological surveillance and control measures but also in association with the rising levels of human migration (9–11).

HAM/TSP development can present distinct speed rates, which have been defined as: (i) very slow, with partial decline of the motor ability after decades of disease; (ii) typical, the most common presentation of the disease; and (iii) rapid progression, characterized by a fast decline of the motor function within two years from symptoms onset (12). Clinical investigation has shown that elevated HTLV-1 proviral load (PVL) is the major factor associated with HAM/TSP development (13–15). However, long-term HTLV-1 asymptomatic carriers (AC) may also present high HTLV-1 PVL, suggesting that this is not the only factor associated with the progression to HAM/TSP (16). Moreover, HTLV-1 PVL has low predictive value as a prognostic biomarker to define the speed of HAM/TSP progression. Therefore, the identification of biomarkers to improve HAM/TSP diagnosis and prognosis has been pursued.

HTLV-1 transmission occurs mainly *via* cell-cell contact and *in vivo* studies report that HTLV-1 preferentially infects CD4^+^ T-cells (17, 18). The precise mechanism of disease development is still not completely clear. However, the most accepted hypothesis is that the inflammatory response of mononuclear cells against infected T-cells within the central nervous system (CNS) indirectly causes the neurological damage (19–21). At the early stages of the disease, infected CD4^+^ T-cells cross the blood-brain barrier (BBB) into the CNS. These cells overproduce interferon-γ (IFN-γ) in HAM/TSP patients, which stimulates CXCL10 secretion by astrocytes. In turn, this process recruits more inflammatory and infected T-cells to sites of active lesions in the spinal cord, thus leading to demyelination, axonal loss, and death of distinct CNS cell types (22).

The presence of elevated levels of inflammatory factors such as neopterin, CXCL10, and CXCL9 in the cerebrospinal fluid (CSF) have been shown to strongly correlate with HAM/TSP development and progression (23–25). Increased CSF levels of interleukin (IL)-1β, IL-6, granulocyte/macrophage-colony stimulating factor (GM-CSF), IFN-γ, and chemokines such as CCL3, CCL5, and CCL11 were also associated with HAM/TSP (26). Furthermore, it was demonstrated that OX40 (CD134), a member of the tumor necrosis factor (TNF) receptor family, was overexpressed in mononuclear cells infiltrating the spinal cord of a patient with early HAM/TSP onset and clinically progressive status. Elevated CSF levels of OX40 were also observed in patients with rapidly progressive HAM/TSP compared to chronic HAM/TSP or other inflammatory neurological diseases (27). Additionally, the vascular cell adhesion molecule 1 (VCAM-1), a protein expressed in the cellular membrane upon stimulation by proinflammatory factors (28, 29) was upregulated in the endothelium of spinal cord lesions (30) and in T-cells from HAM/TSP patients (31). The assessment of the CSF protein profile of HTLV-1-infected individuals also showed that VCAM-1 is differentially expressed in HAM/TSP, and the combination of serum levels of VCAM-1 and SPARC (Secreted protein acidic and rich in cysteine) with the HTLV-1 PVL could predict disease onset in 89.7% of the cases (32).

The aim of this study was to investigate the CSF protein profile in a cohort of HTLV-1 AC and HAM/TSP patients with distinct rates of disease progression to discover biomarkers to improve the diagnosis of HAM/TSP. We show that chitotriosidase 1 (CHIT1) levels in the CSF were strongly associated with the speed of HAM/TSP progression and it could discriminate patients with rapid disease progression. Furthermore, we discuss the association of our findings with other important markers involved in neuroinflammation, neuronal injury, and migration of immune cells to the CNS.

## 2 Material and methods

### 2.1 Study design and population

This is a cross-sectional study performed with paired CSF and serum samples from HTLV-1-infected patients (n=34) older than 18 years enrolled between August 2017 and August 2018 from the cohort of the Instituto Nacional de Infectologia Evandro Chagas (INI) of the Fundação Oswaldo Cruz (FIOCRUZ), Rio de Janeiro, Brazil. This study had the approval of the institutional committee of ethics in research (protocol number 27057119.9.0000.5262, February 6th, 2020). Clinical diagnosis of HAM/TSP was performed following the World Health Organization guidelines (33). Patients were excluded when: diagnosed with co-infection with other viruses (HIV, HBV, HCV and HTLV-2); underwent treatment with corticosteroids or other immunomodulating drugs in 1 year prior to lumbar puncture; diagnosed with ATLL, autoimmune diseases or other chronic inflammatory disorders; diagnosed with other diseases that impair the motor function, such as Parkinson’s syndrome, rheumatoid arthritis, ankylosing spondylitis and others; or presented decubitus bedsores in the six months prior to sample collection.

Study participants were distributed in groups as HTLV-1 AC (n=13) and HAM/TSP patients (n=21). Patients with HAM/TSP were further subdivided according to the rate of disease progression, as previously described (25). Briefly, HAM/TSP patients were evaluated with the IPEC-2 disability scale (**Supplementary Table 1**), which was performed by at least one of the four trained and experienced neurologists who participated in this study, and the disease progression index was obtained by the quotient between the scores and the time of disease in years. The speed of HAM/TSP progression was characterized as: very slow (n=6), for disease progression index ≤ 0.37 points/year; typical (n=9), for index values between 0.38 and 1.44 points/year; and rapid (n=6), for index ≥ 1.45 points/year. Paired CSF (n=9) and serum samples (n=5) from HTLV-1 seronegative individuals with non-inflammatory and non-infectious neurological diseases (normobaric hydrocephalus) were included as negative controls.

### 2.2 Proteomic analysis

#### 2.2.1 Sample preparation and protein digestion

CSF samples from HTLV-1-infected patients were obtained by lumbar puncture between L3/L4 or L4/L5 vertebrates, collected into polypropylene tubes and maintained on ice bath. Aliquots were separated for cell counting, and for the quantification of total proteins and glucose. After, samples were centrifugated at 400 × g for 10 minutes, the supernatants were filtered with 0.45 μm polyethersulfone hydrophilic membrane and stored at -80°C.

At the time of use, CSF samples were thawed and a volume corresponding to 100 μg of total proteins was concentrated in a SpeedVac centrifuge (Thermo Fisher Scientific, MA, USA). To enhance the sensitivity of the proteomic analysis, albumin and immunoglobulin G (IgG), two of the most abundant proteins in the CSF, were depleted using the Pierce™ Albumin/IgG Removal kit (Thermo Fisher Scientific, MA, USA) according to the manufacturer’s instructions. The albumin/IgG-depleted samples were again concentrated in a SpeedVac and resuspended in 20 μL of 0.4 M ammonium bicarbonate and 8 M urea. After, 5 μL of 100 mM dithiothreitol (DTT) was added, and the solution was incubated at 37°C for 1 hour. Following, 5 μL of 400 mM iodoacetamide was added and the solution was further incubated at room temperature and protected from light for 1 hour. Finally, urea concentration was adjusted to 1 M with 130 μL of deionized water (Milli-Q), and protein digestion was performed by overnight incubation at 37°C with 1/50 (m/m) of sequencing grade modified trypsin (Promega, WI, USA).

#### 2.2.2 Liquid chromatography-tandem mass spectrometry analysis

After protein digestion, peptides were desalted in C18 spin-columns and eluted in 0.1% formic acid 40% acetonitrile. Peptides samples were dried in SpeedVac centrifuge without heating and resuspended in 0.1% formic acid 5% DMSO 5% acetonitrile prior to separation by liquid chromatography with the UltiMate™ 3000 RSLCnano System (Thermo Fisher Scientific, MA, USA). The flow was adjusted to 250 nL/min with a 120 min linear gradient of 5−40% acetonitrile and carried out in two phases: (a) 0.1% formic acid, and (b) 0.1% formic acid and 95% acetonitrile. Also, it was used a 15 cm long and 75 μm diameter analytic column containing 3 μm diameter C18 particles and heated at 60°C. Peptides were analyzed in technical duplicates using 600 ng in a Orbitrap Fusion™ Lumos™ Tribrid™ Mass Spectrometer (Thermo Fisher Scientific, MA, USA). For the Ion Source, the spray voltage was set at 2300 V and the ion transfer tube temperature at 175°C. For the Master Scan (MS1), it was used a resolution of 120,000, a *m/z* scan range of 300−1500, the standard AGC Target, an injection time of 50 milliseconds, and it was performed with the incorporation of the EASY-IC™. Additionally, data-dependent acquisition mode was performed to obtain MS2 spectra using an intensity threshold set at 2.0 × 10^4^, an injection time of 22 milliseconds, a dynamic exclusion period of 60 seconds, and a cycle time of 2 seconds between MS1 scans. The higher-energy collisional dissociation with Orbitrap detection (HCD-OT) was implemented for peptide fragmentation and spectra detection, which was performed with the standard AGC Target and the Orbitrap resolution set as 15,000. Each sample was analyzed in two technical runs. The raw data were deposited at the ProteomeXchange Consortium *via* PRIDE under the identification PXD034065.

#### 2.2.3 Data analysis

Mass spectrometry output data were analyzed with the MaxQuant software version 1.6.17.0 (34). The *Homo sapiens* UniProt database (35) (75777 entries of non-redundant, canonical proteins and isoforms, accessed on November 11th 2021) was used for protein identification. Proteins with at least seven amino acid length were accepted while peptide and protein FDR were set at 1%. The oxidation of methionine and acetylation of protein N-terminal modifications were included as variable and carbamidomethylation of cysteine as fixed modification, and both were considered for protein quantification. The resulting summary table with protein identification and intensity values was analyzed with the *R* software version 3.6.1. Proteins representing potential contaminants or identified only by peptides with modified sites were excluded.

Proteins displaying intensity > 0 were considered as identified. Proteins identified in at least one sample were visualized with the *VennDiagram* package and considered for qualitative analysis. The functional enrichment analysis using Gene Ontology (GO) terms of biological process and the Kyoto Encyclopedia of Gene and Genomes (KEGG) databases was performed with the *gprofiler2* package, and the top hits were displayed in dot plot graphs generated by the *enrichplot* package. For the quantitative analysis, the intensity values scale and normalization were carried out with the Perseus software version 1.6.10.43 (36). For this, proteins identified in at least one sample of each group were used. The intensity values were log_2_-transformed, and the missing values were inputted with values from a normal distribution and then normalized by subtracting each value from the median protein intensity among samples. The resulting data matrix was exported and further analyzed in the *R* software.

### 2.3 ELISA

Soluble VCAM-1 (sVCAM-1), chitotriosidase 1 (CHIT1), and cathepsin C (CTSC) were quantified by ELISA using the following kits (all from Thermo Fisher Scientific): Human sVCAM-1 ELISA (Cat. No. BMS232), Human Chitotriosidase ELISA (Cat. No. EH105RB), and Human Cathepsin C ELISA kit (Cat. No. EH73RB). Assays were performed following the manufacturer’s instructions. For sVCAM-1 quantification, CSF and serum samples were diluted at 1:2 and 1:50, respectively. The lower limit of detection (LLOD) for sVCAM-1 was 0.66 ng/mL in the CSF and 16.5 ng/mL in the serum. Quantification of CHIT1 was performed with CSF and serum samples diluted at 1:10 and 1:80, respectively, with a LLOD of 0.04 ng/mL in the CSF and of 0.32 ng/mL in the serum. CSF samples were used undiluted for CTSC quantification, while serum samples were diluted at 1:2, following the manufacturer’s protocol. The LLOD for CTSC in the CSF and serum were 0.95 ng/mL and 2.10 ng/mL, respectively.

### 2.4 Statistical analysis

The analysis of differential protein expression was performed with the Student’s *t*-test. In order to assess candidate biomarkers, correlation analysis was performed between the LC-MS/MS protein intensity and the HAM/TSP progression index, with value 0 attributed to HTLV-1 AC, and significant results were ranked by the *p*-value. After, the protein intensity of potential biomarkers was compared between the groups of HTLV-1 AC and HAM/TSP patients with very slow, typical, and rapid progression using the Kruskal-Wallis test, followed by Dunn’s posthoc test. CSF and serum levels of sVCAM-1, CHIT1, and CTSC defined by ELISA were log_10_-transformed and differences between groups were evaluated with the Kruskal-Wallis test, followed by Dunn’s posthoc test. Unadjusted *p*-values were used and differences with *p* < 0.05 were considered significant. Spearman’s rank of correlation analysis was used to determine the association between distinct CSF markers. Volcano and strip plots were generated with the *ggplot2* package, and scatter plots were constructed with the *ggpubr* package for *R* software.

## 3 Results

### 3.1 CSF protein profile in HTLV-1-infected individuals

**Figure 1:**
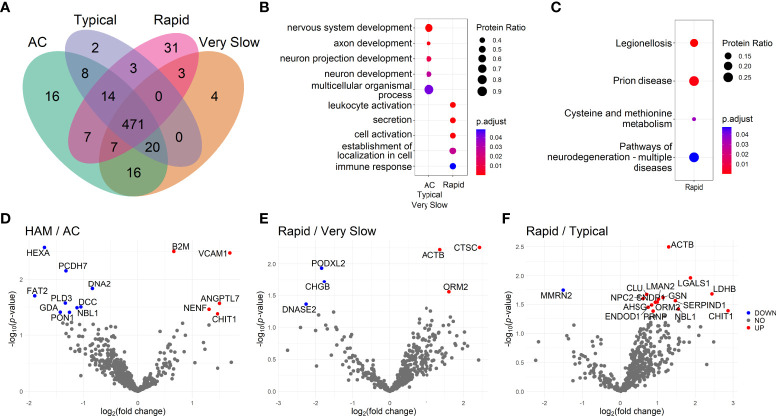
Investigation of the cerebrospinal fluid proteome of HTLV-1-infected individuals. **(A)** Venn diagram shows the number of proteins identified by LC-MS/MS in samples from patients separated as HTLV-1 asymptomatic carriers (AC) (n=13), and HAM/TSP patients with very slow (n=6), typical (n=7), and rapid (n=6) disease progression. Functional enrichment analysis of protein sets was performed using the databases from **(B)** the Gene Ontology terms of biological processes and **(C)** the Kyoto Encyclopedia of Genes and Genomes (KEGG). The size of the dots is proportional to the number of proteins enriched in the process/pathway, and the color gradient indicates the adjusted *p*-values. **(D–F)** Differential protein expression between groups according to the neurological condition is presented in Volcano plots showing uncorrected -log_10_-transformed *p*-values and the log_2_-transformed fold change of common proteins between the two groups. Proteins differentially expressed with log_2_-transformed fold change higher than 0.6 and lower than -0.6 and were considered upregulated (red dots) and downregulated (blue dots), respectively. The statistical analysis was performed with the Student’s *t*-test. Differences with -log_10_-transformed *p*-value higher than 1.3 were considered significant.

The CSF protein profile was determined in samples from HTLV-1 AC and HAM/TSP patients. In total, 602 proteins were identified and the distribution among groups is displayed in the Venn diagram (**Figure 1A**) (**Supplementary Table 2**). HAM/TSP patients were subdivided according to the speed of disease progression. HTLV-1 AC and HAM/TSP patients showed a similar profile, with 543 proteins in common (90.2%). It was observed that 16 proteins were exclusively identified in HTLV-1 AC and 43 proteins were detected only in HAM/TSP patients, and 31 of them (72.1%) were associated with rapid disease progression.

The functional enrichment analysis was performed with protein sets identified in each group of patients. The top hits associated with protein sets from selected groups are displayed in **Figures 1B, C**. HAM/TSP with rapid progression presented an association with leukocyte activation (GO:0045321, 13 of 31 proteins), cell activation (GO:0001775, 13 of 31 proteins) and immune response processes (GO:0006955, 14 of 31 proteins). The analysis using the KEGG database also revealed the stimulation of neurodegeneration pathways (KEGG:05022, 7 of 27 proteins). Among HTLV-1 AC, proteins exclusively detected in this group were related to positive regulation of apoptotic cell clearance (GO:2000427, 2 of 15 proteins) (**Supplementary Table 3**). Conversely, the small number of proteins exclusively identified in the groups of HAM/TSP patients with typical and very slow disease progression limited their analysis. The evaluation of proteins found in the intersection between HTLV-1 AC and HAM/TSP patients with typical and very slow disease progression, that is excluding only rapid progressors, displayed an association with processes of nervous system development (GO:0007399, 13 of 20 proteins), including neuron development, projections and axon development (**Figure 1B**). In contrast, the analysis using the KEGG database returned no hits for proteins found in the groups of HTLV-1 AC and HAM/TSP patients with typical and very slow progression as well as in their intersections (**Supplementary Table 3**).

The differential expression analysis showed that the soluble vascular cell adhesion molecule 1 (sVCAM-1) was the most upregulated protein in HAM/TSP patients compared to HTLV-1 AC (log_2_ fold-change = 1.696, *p* = 0.004) (**Figure 1D**). In addition, the chitotriosidase 1 (CHIT1) protein was also upregulated in HAM/TSP patients (log_2_ fold-change = 1.467, *p* =0.042) (**Figure 1D**). Furthermore, HAM/TSP rapid progressors had increased cathepsin C (CTSC) expression compared to individuals with very slow disease progression (log_2_ fold-change = 2.431, *p* = 0.006) (**Figure 1E**). Moreover, the differential protein expression between individuals with rapid and typical HAM/TSP progression revealed that CHIT1 (log_2_ fold-change = 2.865, *p* = 0.041) was the most upregulated protein among rapid progressors (**Figure 1F**). The statistical analysis of CHIT1 expression in HAM/TSP patients with very slow progression was not possible since no intensity for this protein was detected in this group. In addition, the comparison between individuals with typical and very slow disease progression resulted in very few and unremarkable differentially expressed proteins (**Supplementary Figure 1**).

The functional enrichment analysis of upregulated proteins in HAM/TSP patients with rapid progression reinforced the relevance of the inflammatory process. In the comparison with the groups of HTLV-1 AC, and HAM/TSP patients with typical and very slow progression, it was respectively observed enriched processes of positive regulation of immune system process (GO:0002684, 7 of 11 proteins), leukocyte activation (GO:00045321, 8 of 15 proteins), and apoptosis (KEGG:04210, 2 of 2 proteins). On the other hand, processes of angiogenesis (GO:0001525, 3 of 3 proteins) were enriched in HTLV-1 AC compared to HAM/TSP rapid progressors (**Supplementary Table 4**).

### 3.2 CSF and serum levels of sVCAM-1, CHIT1, and CTSC

To assess candidate biomarkers, it was performed the correlation analysis between the intensity of all proteins identified by LC-MS/MS and the HAM/TSP progression index. Statistically significant results were ranked by the *p*-value. CHIT1 (*R* = 0.587, *p* < 0.001) and beta-2-microglobulin (B2M; *R* = 0.515, *p* = 0.003) showed the most significant correlations (**Table 1**). In HAM/TSP rapid progressors, CHIT1 and CTSC displayed higher intensity in LC-MS/MS compared to HTLV-1 AC and HAM/TSP patients with very slow progression (**Supplementary Figure 2**). Furthermore, higher protein intensity in HAM/TSP rapid progressors was also observed for B2M, sVCAM1, ANGPTL7, and COL1A1 compared to HTLV-1 AC (**Supplementary Figure 2**). Indeed, the differential protein expression analysis revealed a greater log_2_ fold-change for sVCAM1, CHIT1, and CTSC among HAM/TSP patients with rapid progression (**Figures 1E, F** and **Supplementary Figure 1**), which determined the choice of these three proteins for further validation by ELISA.

**Table 1:** Correlation between the LC-MS/MS protein intensity and the HAM/TSP progression index.

Protein name	Uniprot accession	Spearman *R*	*p*-value
**CHIT1**	Q13231	0.587	<0.001
**B2M**	P61769	0.515	0.003
**PCDHAC2**	Q9Y5I4	-0.470	0.007
**HEXA**	P06865	-0.468	0.007
**CANT1**	Q8WVQ1	-0.445	0.011
**PLXDC1**	Q8IUK5	-0.435	0.013
**ALDOA**	P04075	-0.430	0.014
**MRC1**	P22897	0.427	0.015
**VCAM1**	P19320	0.427	0.015
**LYVE1**	Q9Y5Y7	-0.407	0.021
**ANGPTL7**	O43827	0.398	0.024
**ENO2**	P09104	-0.389	0.028
**IDS**	P22304	-0.380	0.039
**DNA2**	P51530	-0.377	0.033
**COL1A1**	P02452	0.375	0.034
**CTSC**	P53634	0.368	0.038
**MAN2A2**	P49641	-0.361	0.043
**SPARC**	P09486	-0.358	0.044
**CDH5**	P33151	-0.356	0.046
**ATRN**	O75882	-0.354	0.046

**Figure 2:**
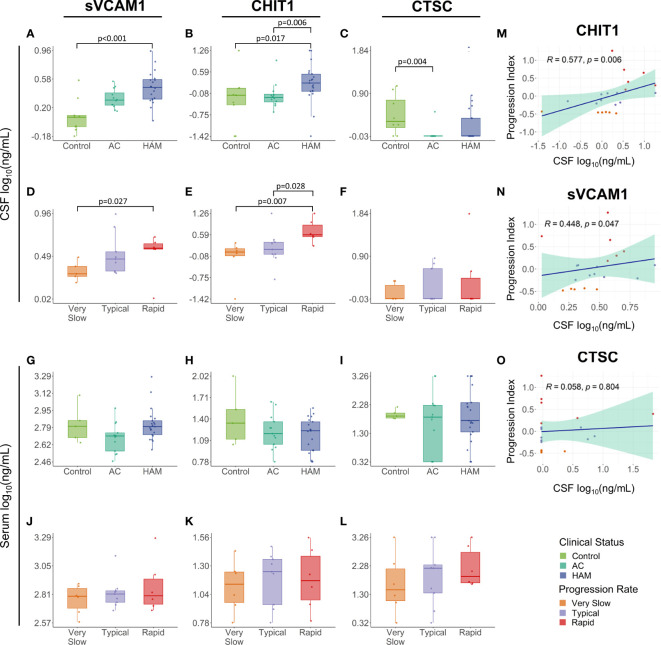
Validation of proteome findings in the cerebrospinal fluid (CSF). sVCAM-1, chitotriosidase 1 (CHIT1), and cathepsin C (CTSC) concentration were determined by ELISA in paired **(A–F)** CSF and **(G–L)** serum samples from HTLV-1-infected individuals identified according to their clinical status as HTLV-1 asymptomatic carriers (AC) (n=13) and HAM/TSP patients (n=21). The CSF (n=9) and serum samples (n=5) from individuals with non-inflammatory and non-infectious neurological diseases were used as a control. HAM/TSP patients were also subdivided according to the speed of disease progression as very slow (n=6), typical (n=9) and rapid (n=6). The statistical analysis was performed with Kruskal-Wallis test, followed by Dunn’s posthoc test. Unadjusted *p*-values were used and differences with *p* < 0.05 were considered significant. **(M–O)** The association of the HAM/TSP progression index, defined by the speed of neurological deterioration in the IPEC-2 disability scale and the time of disease, and the CSF levels of CHIT1, sVCAM-1, and CTSC was evaluated by Spearman’s rank of correlation analysis, and *p*-values < 0.05 were considered significant.

The concentrations of these proteins were determined in paired CSF and serum samples from HTLV-1-infected individuals and an HTLV-1 seronegative control group. sVCAM-1 levels were higher in the CSF of HAM/TSP patients (2.94 ng/mL, IQR 2.06-3.74 ng/mL) in comparison with the control group (1.19 ng/mL, IQR 0.89-1.25 ng/mL) (Dunn’s test *p* < 0.001). HAM/TSP patients also displayed increased CHIT1 CSF levels (1.74 ng/mL IQR, 0.97-3.32 ng/mL) compared to the HTLV-1 AC (0.62 ng/mL, IQR 0.45-0.79 ng/mL) and control groups (0.73 ng/mL, IQR 0.36-1.21 ng/mL) (Dunn’s posthoc test *p* = 0.006 and 0.017, respectively) (**Figures 2A, B**). In turn, CTSC levels in the CSF were higher in uninfected individuals (1.99 ng/mL, IQR 1.49-6.05 ng/mL) than in HTLV-1 AC, which presented values below the LLOD of the assay (< 0.95 ng/mL) (Dunn’s test *p* = 0.004) (**Figure 2C**). HAM/TSP patients were also evaluated according to the speed of disease progression. Rapid progressors showed significantly higher CSF levels of sVCAM-1 (3.78 ng/mL, IQR 3.69-4.24 ng/mL) and CHIT1 (3.88 ng/mL, IQR 3.40-8.24 ng/mL) compared to very slow progressors (sVCAM-1: 1.98 ng/mL, IQR 1.86-2.39 ng/mL; and CHIT1: 1.13 ng/mL, IQR 0.86-1.44 ng/mL) (Dunn’s test *p* = 0.0274 and 0.0070, respectively) (**Figures 2D, E**). Moreover, HAM/TSP patients with rapid disease progression also had higher CHIT1 CSF levels compared to those with typical progression (1.35 ng/mL, IQR 0.96-2.28 ng/mL) (Dunn’s test *p* = 0.0284) (**Figure 2E**). Furthermore, no difference was also observed in CTSC concentration between groups (**Figure 2F**). Conversely, these proteins did not show altered serum levels between groups (**Figures 2G–L**).

Interestingly, CSF levels of sVCAM-1 and CHIT1 slightly increased with the rate of disease progression, as observed in **Figures 2D, E**. Indeed, correlation analysis confirmed an association between the HAM/TSP progression index and the CSF concentration of CHIT1 (Spearman R = 0.577, p = 0.006) and sVCAM-1 (Spearman R = 0.448, p = 0.047) (**Figures 2M, N**) but not for CTSC (Spearman R = 0.058, p = 0.804) (**Figures 2O**). Moreover, the reliability of the proteome profile determined by LC-MS/MS analysis was confirmed by multiple regression analysis using data of the CSF CHIT1 levels quantified by ELISA and the CSF CHIT1 intensity, which showed a significant correlation (*R* ^2^ = 0.662, *p* < 0.001) (**Supplementary Figure 3**).

### 3.3 CHIT1 and sVCAM-1 levels correlate with biomarkers of neuroinflammation and neurodegeneration in HAM/TSP patients

**Figure 3:**
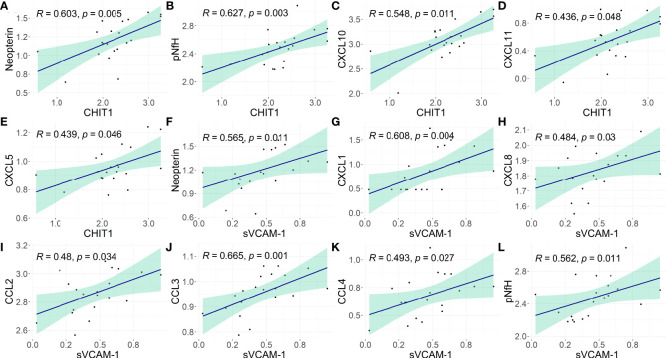
Association of CHIT1 and sVCAM-1 with biomarkers of neuroinflammation and neuronal damage in the CSF of HAM/TSP patients. The correlation between the CSF concentration of **(A–E)** CHIT1 and **(F–L)** sVCAM-1 with neopterin, phosphorylated neurofilament heavy chain (pNfH) protein, and proinflammatory chemokines (CCL2, CCL3, CCL4, CXCL1, CXCL5, CXCL8, CXCL10, and CXCL11) was performed with Spearman’s rank of correlations test. The biomarkers of neuroinflammation or neuronal damage were assessed in this population in a previous study (25). The concentration values were log_2_-transformed and *p*-values <0.05 were considered significant.

Distinct biomarkers of neuroinflammation and neuronal death were previously evaluated in this study population (25). In HAM/TSP patients, CSF levels of CHIT1 positively correlated with neopterin, a byproduct from the monocyte/macrophage inflammatory response (Spearman *R* = 0.603, *p* = 0.005), and with the phosphorylated neurofilament heavy protein (pNfH), a biomarker for neuronal damage (Spearman *R* = 0.627, *p* = 0.003). It was also observed a positive correlation with chemokines in the CSF, and increasing CSF CHIT-1 levels were associated with elevated levels of CXCL10 (Spearman *R* = 0.548, *p* = 0.011) and CXCL11 (Spearman *R* = 0.436, *p* = 0.048), which are potent chemoattractants to Th1 cells, and with CXCL5 levels (Spearman *R* = 0.439, *p* = 0.046) (**Figures 3A−E** and **Supplementary Table 5**).

In HAM/TSP patients, CSF levels of sVCAM-1 positively correlated with CSF neopterin (Spearman *R* = 0.565, *p* = 0.011). A positive correlation was also observed with CXCL1 and CXCL8 (Spearman *R* = 0.608, *p* = 0.004; and Spearman *R* = 0.484, *p* = 0.03, respectively), which are involved in the recruitment of neutrophils and NK cells, with CCL2 (Spearman *R* = 0.48, *p* = 0.034), CCL3 (Spearman *R* = 0.665, *p* = 0.001), and CCL4 (Spearman *R* = 0.493, *p* = 0.027), which are chemoattractant to monocytes and T-cells. In addition, CSF sVCAM-1 levels also correlated with the CSF concentration of pNfH in HAM/TSP patients (Spearman *R* = 0.562, *p* = 0.011) (**Figures 3F−L**; **Supplementary Table 5**).

## 4 Discussion

The study of the protein profile of human tissues and bodily fluids has been an important tool for the discovery of disease biomarkers for early diagnosis or prognosis, particularly in disorders with silent onset or rapid progression. In addition, data from high throughput assays also have great value in identifying biological processes underlying mechanisms of disease development. In this study, the CSF of HAM/TSP patients showed elevated CHIT1 levels even though the CSF of these individuals showed high similarity with HTLV-1 AC, since most proteins identified in the proteomic analysis (90.2%) were shared between groups. Interestingly, when HAM/TSP patients were divided according to the speed of disease progression, 72.1% of the proteins (31 of 43 proteins) identified in this group were exclusively expressed in rapid progressors. This suggests that unique processes have been upregulated in this group as a sign of the fast neurological decline observed.

HAM/TSP patients with rapid progression expressed proteins related to immune response and leukocyte/cell activation, and pathways associated with neurodegeneration. In agreement with our study, rapid progressors seem to present stronger inflammatory activity in the spinal cord (12). Therefore, these data suggest that the speed of HAM/TSP progression might be defined or influenced by unbalanced deleterious and protective responses acting in the processes of neuroinflammation and neurodegeneration. However, further studies are still needed to elucidate this hypothesis.

The CHIT1 is expressed by cells of the myeloid lineage, particularly by mature macrophages, and by microglia cells in the CNS (37, 38). This protein has a chitinase activity and therefore has a role in the immune response to chitin-containing pathogens (39). Furthermore, in the CNS, CHIT1 is associated with adaptive Th2 response, tissue remodeling, and oligodendrogenesis (37). CHIT1 was also shown to promote a TGF-β-induced uptake of β-amyloid by microglia cells *in vitro* (40). To our knowledge, this study is the first to associate CHIT1 expression with HAM/TSP development. Altered levels of CHIT1 in the CSF have been shown as a biomarker for other neurodegenerative disorders, such as Alzheimer’s disease, amyotrophic lateral sclerosis (ALS), and multiple sclerosis (41–43). In ALS, it was observed an association between CSF CHIT1 levels and both disease severity and progression (44, 45). In addition, CHIT1 intrathecal administration in a rat experimental model induced microglia and astrocyte activation leading to neuroinflammation and neuronal loss in the spinal cord (44). Similar findings were observed in patients with multiple sclerosis with rapid neurological deterioration, which displayed higher CSF CHIT1 levels in addition to other markers of microglial activity, such as TREM-2 (triggering receptor expressed on myeloid cells 2) and CH3L1 (chitinase-3-like protein 1 precursor) (41). In this study, the HTLV-1-infected individuals with distinct levels of neurological involvement showed no difference in the serum concentration of CHIT1. This suggests that CHIT1 was intrathecally produced. In ALS, controversial results indicate that only CSF CHIT1 levels or both CSF and serum levels are useful as disease biomarker (43, 44). However, the lack of paired CSF and serum samples in these studies might limited to obtain consistent conclusions.

High CSF sVCAM-1 was also associated with faster HAM/TSP progression in our study, although it was not possible to use this protein as a biomarker to discriminate between HAM/TSP patients and HTLV-1 AC, or to predict the speed of HAM/TSP progression. However, sVCAM-1 also displayed distinct levels in the CSF and the serum of HTLV-1-infected individuals, also suggesting intrathecal production and shedding. In contrast, Ishihara et al. (32) observed an upregulation of sVCAM-1 in the CSF and plasma of unpaired HTLV-1-infected populations. This controversy was also observed in patients with multiple sclerosis, in which elevated sVCAM-1 in the CSF did not consistently correlate with serum/plasma levels (46, 47). Moreover, patients with subacute HIV-associated dementia presented higher levels of sVCAM-1 in CSF but not in serum (48).

The release of sVCAM-1 from brain endothelial cells seems to play a role in inflamed BBB in multiple sclerosis, enhancing the BBB permeability by autocrine stimulation (49). Since sVCAM-1 was not elevated in the serum of HAM/TSP patients, VCAM-1 production and release was likely occurring within the CNS and not by transport through the BBB. The frequency of microglia expressing VCAM-1 is enhanced in oligodendrocyte injured regions in multiple sclerosis, while endothelial cells in spinal cord lesions were VCAM-1 negative, suggesting its participation in the demyelination process (50). Moreover, the CSF patients with HIV-associated neurocognitive disorder show a correlation between increased levels of sVCAM-1 and markers of macrophage/microglia (CD163 and CD14) (48). In HAM/TSP patients, increased expression of VCAM-1 and its ligand (VLA-4) is present in chronically active spinal cord lesions (19). Moreover, the frequency of T-cells expressing CD49d (Integrin subunit alpha 4), a component of VLA-4, is increased in the peripheral blood of HTLV-1-infected individuals (51). Therefore, more studies are needed to investigate whether CNS cells are responsible for VCAM-1 shedding and the role of this protein in processes related to neuronal damage and demyelination in HAM/TSP.

Recently, neopterin and CXCL10 have been shown as promising biomarkers for HAM/TSP progression evaluated with the Osame Motor Disability Score (24), and confirmed in this study population using the IPEC-2 disability scale (25). Neopterin is a byproduct of immune responses mediated by monocytes/macrophages. Indeed, CSF neopterin has been shown as a biomarker for numerous viral and bacterial diseases affecting the CNS as well as in chronic neuroinflammatory disorders (52). In this study, CHIT1 and sVCAM-1 showed a significant correlation with neopterin in the CSF of HAM/TSP patients. In addition, CHIT1 and sVCAM-1 also showed an association with pNfH, a component of neurofilaments and a biomarker for the destruction of myelinated axons. In ALS patients, pNfH has been associated with higher CHIT1 levels of in the CSF (43, 53). The neurofilament light chain (NfL) protein, another biomarker for axonal loss, also correlates with CSF CHIT1 levels in patients with ALS and multiple sclerosis (42, 54). This association however was not observed in our study, likely as a result from the chronic and slow progression rate of HAM/TSP compared to ALS and multiple sclerosis, in addition to the shorter half-life of NfL in comparison to pNfH.

In addition, CHIT1 correlated with the concentration of CXCL5, CXCL10, and CXCL11 in 13 chemokines previously assessed in the CSF of this study population. CXCL10 and CXCL11 are ligands of the CXCR3, a chemokine receptor expressed in Th1 cells and CD8^+^ T-cells and strongly associated with HAM/TSP progression (24, 25). This confirms the importance of CXCR3 chemotactic axis in the recruitment of Th1 and CD8^+^ T-cells to the CNS and in HAM/TSP pathogenesis. In addition, CSF sVCAM-1 correlated with CCL3 and CCL4, ligands of CCR5, a chemokine receptor also present in Th1 and CD8^+^ T-cells as well as in monocytes. These inflammatory chemokines were also upregulated in the CSF of HAM/TSP patients compared to HTLV-1 AC (25), although they were not identified as biomarkers for disease progression. Therefore, the correlation between CSF CHIT1 and the HAM/TSP progression index and CXCL10 levels strengthen the indication of CHIT1 as a candidate biomarker for CXCL10.

CHIT1 and sVCAM-1 concentration in CSF also correlated with chemokines involved in the chemotaxis of polymorphonuclear cells, such as CXCL5, CXCL8, and CXCL1, all ligands of CXCR2. However, cells infiltrating the CNS of HAM/TSP patients were predominantly mononuclear cells (25), as well as previously shown by others (19). Therefore, the CSF concentration of these chemokines in HAM/TSP neuroinflammation might not be sufficient to attract polymorphonuclear cells expressing CXCR2 into the CNS. Nonetheless, activated astrocytes secrete CXCL1, which in turn can induce VCAM-1 expression in cerebral endothelial cells (55). Therefore, the production of these cytokines may represent a side-effect of the strong activation of astrocytes, as shown by their involvement with CXCL10 secretion in response to IFN-γ released by HTLV-1-infected cells (56).

Interestingly, the mass spectrometry analysis suggested that HAM/TSP rapid progressors had higher CTSC levels in the CSF compared to individuals with very slow progression. In the CNS, CTSC promotes microglia M1 polarization (57) and it is highly expressed under neuroinflammatory conditions (58), particularly in areas of demyelination (59). However, it was not possible to determine whether CTSC plays a role in HAM/TSP development since CTSC concentration was undetectable in the CSF of most participants and no difference was observed between groups. Unfortunately, the lower limit of detection of ELISA kits commercially available for the CTCS quantification did not allow to determine its CSF concentration.

In contrast to rapid progressors, HAM/TSP patients with typical and very slow progression presented CSF protein profiles closer to HTLV-1 AC, particularly of proteins related to processes to stabilize and/or to limit the CNS damage. These groups presented the expression of proteins related to nervous system development, which may represent a counter-response to the neuronal damage promoted by the inflammatory response against HTLV-1 infection. These proteins participate in the guidance, synaptic regulation, and survival of regenerating axons, including cell adhesion molecules such as the cell adhesion molecule L1 like protein (CHL1) (60, 61) and the contactin 6 (CNTN6) (62). Other factors are involved in synaptic organization, such as the heterodimer receptor formed by neuropilin 2 (NRP2) and plexinA4 that binds the endothelial-secreted semaphorin-3G (SEMA3G). However, it was shown that SEMA3G is reduced in the CSF of patients with Alzheimer’s disease, which might contribute to the impairment of the structure and the plasticity of synapses (63). In addition, protocadherin alpha-C2 (PCDHAC2), a protein expressed in the axons and synaptic junctions of neurons, is associated to neuronal survival, synaptic connectivity, axonal convergence, and axonal projection of serotoninergic neurons (64). The αGDP dissociation inhibitor 1 protein (coded by *GDI1* gene) is another protein participating in synaptic organization. GDI1 regulates the rab GTPase cycle and therefore can modulate the intracellular protein traffic, and the absence of CDI1 in the brain leads to the impairment of pre-synaptic functions and synaptic plasticity (65). Other proteins are associated with prevention of neurotoxicity, such as the arylsulfatase A (ARSA), an intracellular enzyme that hydrolyzes cerebroside sulfate, which is a lipid abundant in myelin that is neurotoxic when accumulated. Therefore, ARSA activity protects the CNS cells and prevents microglial activation and neuroinflammation (66). Thus, it is possible that these processes of neuronal development, plasticity, and survival might contribute to the prevention of HAM/TSP development or to slow down the speed of disease progression.

This study presented limitations, particularly regarding to the small sample size and the lack of a distinct testing population. However, the conduction of long-term cohort studies, with a structured neurological follow-up to determine the speed of HAM/TSP progression, and the recovery of paired CSF and serum samples are quite challenging. Despite that, our findings did demonstrate that CHIT1 levels in the CSF represent a promising biomarker for the prognosis of HAM/TSP, particularly to differentiate patients with rapid progression and worse prognosis. Since CHIT1 plays a role in proinflammatory responses observed in other neurodegenerative disorders, it would be interesting to investigate whether CHIT1 expression by CNS-infiltrating macrophages and/or resident microglial cells influence the positive regulatory feedback of neuroinflammation, and thus increasing the neurodegeneration in HAM/TSP.

## Data availability statement

The data presented in the study are deposited in the Proteomics Identifications Database (PRIDE) repository (https://www.ebi.ac.uk/pride), accession number PXD034065.

## Ethics statement

This study was reviewed and approved by the committee of ethics in research of the Evandro Chagas National Institute of Infectious Diseases (INI) of the Oswaldo Cruz Foundation (FIOCRUZ). The patients/participants provided their written informed consent to participate in this study.

## Author contribution

OME designed the study. YG, NF, FS, and OME performed assays. YG, VS, DP, FN carried out data analysis. AL, ML, MS and AA conducted the neurological assessment and clinical follow-up of patients. YG carried out the statistical analysis. YG, JE-L, AV and OME drafted the manuscript. All authors contributed to the article and approved the submitted version.

## Funding

OME was awarded with grants in the Programa Jovens Pesquisadores/INI - Fundação Oswaldo Cruz (Grant number INI-003-FIO-19-2-14) and the INOVA Program - Fundação Oswaldo Cruz (Grant number VPPCB-008-FIO-18-2-39). YCPG was granted with a PhD scholarship from the Coordination for the Improvement of Higher Education Personnel (CAPES) of the Brazilian Ministry of Education, and NLF was granted with a Master’s scholarship from the Fundação Oswaldo Cruz.

## Acknowledgments

We thank the Program for Technological Development in Tools for Health-PDTIS FIOCRUZ for the permission to use of its facilities. We also thank Dr. Michel Batista and all members of the Mass Spectrometry Facility at FIOCRUZ-Paraná for LC-MS/MS data acquisition and the identification of proteins.

## Conflict of interest

The authors declare that the research was conducted in the absence of any commercial or financial relationships that could be construed as a potential conflict of interest.

## Publisher’s note

All claims expressed in this article are solely those of the authors and do not necessarily represent those of their affiliated organizations, or those of the publisher, the editors and the reviewers. Any product that may be evaluated in this article, or claim that may be made by its manufacturer, is not guaranteed or endorsed by the publisher.
