# Supplementary material for: Chitotriosidase 1 in the cerebrospinal fluid as a putative biomarker for HTLV-1-associated myelopathy/tropical spastic paraparesis (HAM/TSP) progression
Source: Front Immunol. 2022 Aug 16;13:949516. doi: 10.3389/fimmu.2022.949516 (PMC9424492; doi:10.3389/fimmu.2022.949516)
Supplement: Supplementary file 1 [file DataSheet_1.pdf]

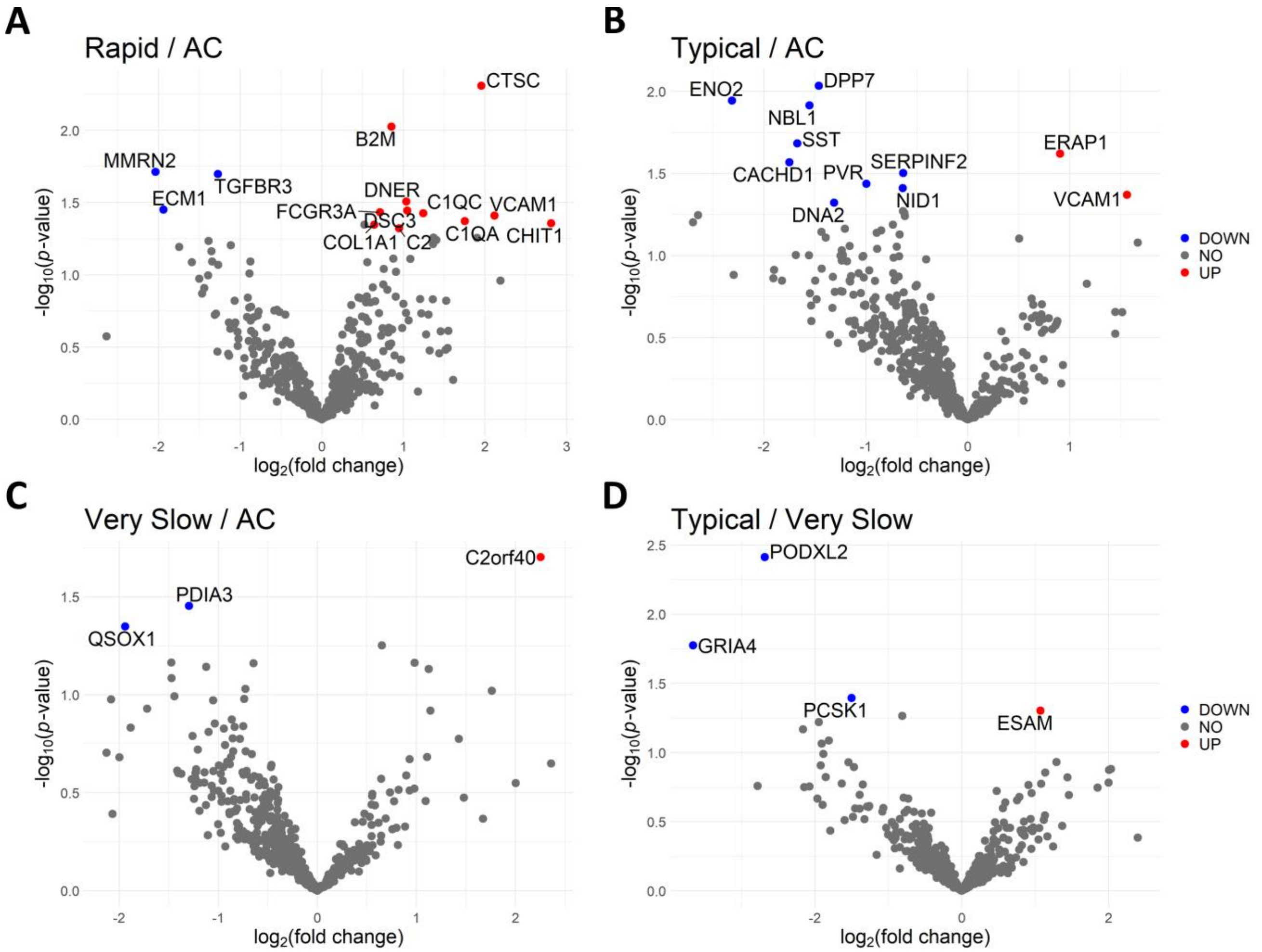

Supplementary Figure 1. Differential protein expression analysis of the cerebrospinal fluid from HTLV-1-infected individuals. Proteins were identified by LC-MS/MS analysis. Volcano plots show the uncorrected -$\log_{10}$-transformed *p*-values and the $\log_2$-transformed fold change of common proteins between the groups of **(A)** HTLV-1 asymptomatic carriers (AC) and HAM/TSP patients with rapid progression, **(B)** HTLV-1 AC and HAM/TSP patients with typical progression, **(C)** HTLV-1 AC and HAM/TSP patients with very slow progression, and **(D)** HAM/TSP patients with typical and very slow progression. Proteins differentially expressed with $\log_2$-transformed fold change higher than 0.6 and lower than -0.6 and were considered upregulated (red dots) and downregulated (blue dots), respectively. The statistical analysis was performed with the Student's *t*-test. Differences with -$\log_{10}$-transformed *p*-value higher than 1.3 were considered significant.
