# Supplementary material for: Chitotriosidase 1 in the cerebrospinal fluid as a putative biomarker for HTLV-1-associated myelopathy/tropical spastic paraparesis (HAM/TSP) progression
Source: Front Immunol. 2022 Aug 16;13:949516. doi: 10.3389/fimmu.2022.949516 (PMC9424492; doi:10.3389/fimmu.2022.949516)
Supplement: Supplementary file 2 [file DataSheet_2.pdf]

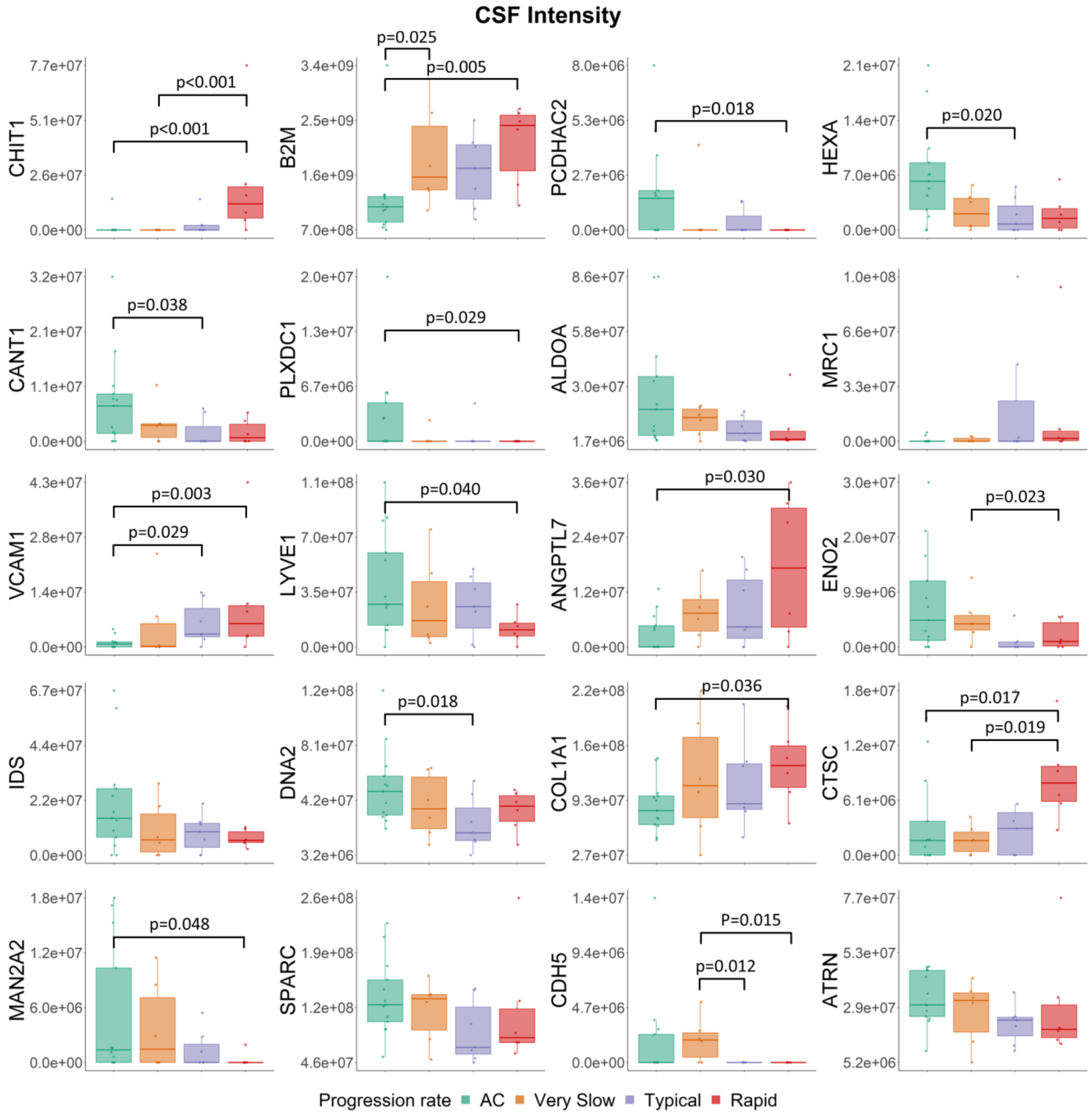

Supplementary Figure 2. Comparison of the LC-MS/MS intensity of the 20 proteins in the cerebrospinal fluid (CSF) with significant correlation with the HAM/TSP progression index. Patients were identified according to their clinical status as HTLV-1 asymptomatic carriers (AC) (n=13), HAM/TSP patients with very slow (n=6), typical (n=9), and rapid (n=6) progression. The statistical analysis was performed with Kruskal-Wallis test, followed by Dunn's posthoc test. Unadjusted *p*-values were used and differences with $p < 0.05$ were considered significant.
