# Supplementary material for: Chitotriosidase 1 in the cerebrospinal fluid as a putative biomarker for HTLV-1-associated myelopathy/tropical spastic paraparesis (HAM/TSP) progression
Source: Front Immunol. 2022 Aug 16;13:949516. doi: 10.3389/fimmu.2022.949516 (PMC9424492; doi:10.3389/fimmu.2022.949516)
Supplement: Supplementary file 3 [file DataSheet_3.pdf]

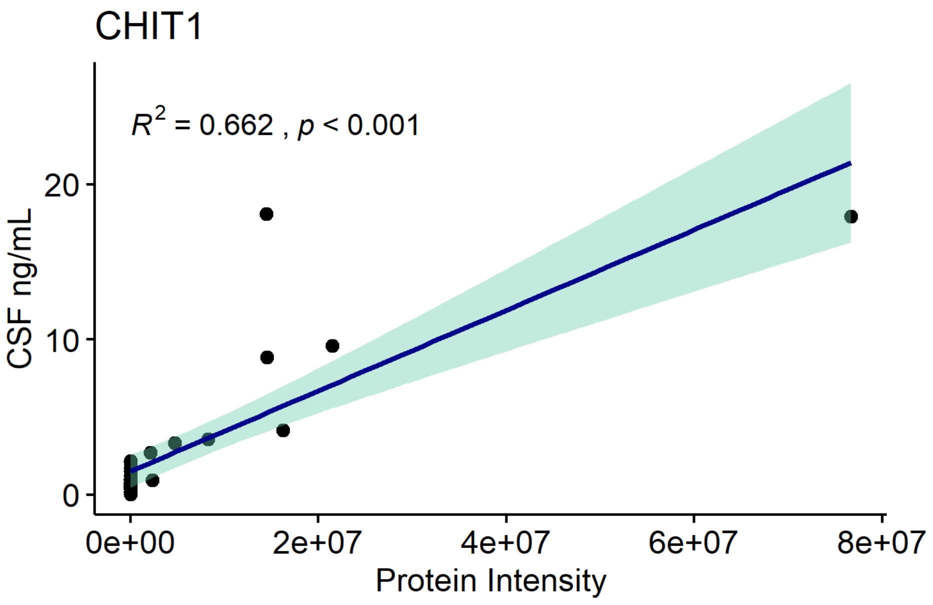

Supplementary Figure 3. Association between CHIT1 intensity in LC-MS/MS and CHIT levels defined by ELISA. Multiple regression analysis was performed and *p*-value <0.05 was considered significant.
