# Supplementary material for: Chitotriosidase 1 in the cerebrospinal fluid as a putative biomarker for HTLV-1-associated myelopathy/tropical spastic paraparesis (HAM/TSP) progression
Source: Front Immunol. 2022 Aug 16;13:949516. doi: 10.3389/fimmu.2022.949516 (PMC9424492; doi:10.3389/fimmu.2022.949516)
Supplement: Supplementary file 4 [file Table_1.pdf]

frontiers

Supplementary Table 1. IPEC-2 disability scale.

Motor score: Gait
0. Normal
1. Abnormal but can walk independently
2. Abnormal and dependent on eventual unilateral support
3. Abnormal and dependent on permanent unilateral support
4. Abnormal and dependent on eventual bilateral support
5. Abnormal and dependent on permanent bilateral support
6. Abnormal, dependent on permanent bilateral support, and occasional use of a wheelchair (WC)
7. Permanent use of a WC, stands up and remains upright without support
8. Permanent use of a WC, uses arms to stand up and remains upright without support
9. Permanent use of a WC, needs assistance from others to stand up and remain upright with support
10. Permanent use of a WC, unable to stand up, exhibits voluntary movements of the lower limbs when seated
11. Permanent use of WC, unable to stand up, and does not have any voluntary movements of the lower limbs

Motor score: Running
0. Runs
1. Unable to run

Motor score: Climbing stairs
0. Climbs
1. Climbs only when holding the handrail
2. Unable to climb

Motor score: Jumping
0. Jumps on one or two feet
1. Jumps on two feet, but not with only one
2. Jumps on two feet only with hand support
3. Unable to jump

Spasticity score: Clonus
0. Absent
1. Only induced by the examiner
2. Spontaneous

Spasticity score: Flexor/extensor spasms
0. Absent
1. Present

Sensory score: Paresthesia
0. Absent
1. Present, eventually
2. Present, permanently

Sensory score: Lumbar pain
0. Absent
1. Present, eventually
2. Present during most of the day

Sensory score: Lower limb pain
0. Absent
1. Present, eventually
2. Present during most of the day

Sphincter score: Bladder control
0. Total
1. Urgency
2. Eventual incontinence or retention
3. Use of permanent catheter or regular use of relieving catheter

Sphincter score: Bowel continence
0. Normal
1. Constipation
2. Incontinence or total retention, needs manual extraction or enemas

Total: 0–31

From Souza et al., Frontiers in Immunology 2021;12:737941 (doi: 10.3389/fimmu.2021.737941)
